# Shoulder dislocation in patients older than 60 years of age

**DOI:** 10.4103/0973-6042.79792

**Published:** 2010

**Authors:** Jose M. Rapariz, Silvia Martin-Martin, Antonio Pareja-Bezares, Jose Ortega-Klein

**Affiliations:** Department of Orthopedic Surgery, Hospital Son Llàtzer, Palma de Mallorca, Spain; 1 Department of Radiology, Hospital Son Llàtzer, Palma de Mallorca, Spain; 2 Department of Epidemiology, Hospital Son Llàtzer, Palma de Mallorca, Spain

**Keywords:** Shoulder dislocation, shoulder instability, elderly

## Abstract

**Purpose:**

Recurrent anterior shoulder dislocation in elderly patients is a little studied condition. The goal of this paper is to clarify the role of associated injuries with respect to loss of function and recurrence of dislocation.

**Materials and Methods:**

We have conducted a retrospective, descriptive study on 29 patients older than 60 years at the moment they suffered their first dislocation episode. All patients were assessed clinically (Constant test) and by imaging testing (X-ray, MRI).

**Results:**

Nine (31.03%) out of 29 patients had a recurrent dislocation. Four of them required reconstructive surgery to maintain joint stability. Injury to the anterior support (anterior labrum, anterior glenoid rim) showed a statistically significant relation to the recurrence of dislocations. The occurrence or non-occurrence of a rotator cuff tear does have an impact on the shoulder function. The degree of rotator cuff involvement on the coronal plane does not significantly affect the shoulder’s functional outcome. The tear extension on the sagittal plane does cause impairment on the Constant test.

**Conclusions:**

Labrum and/or anterior glenoid involvement should be suspected in elderly patients presenting with recurrent shoulder dislocation. Recurrence is due to an injury in the anterior support or both (anterior and posterior), even though shoulder function gets impaired when a rotation cuff tear occurs with anterior extension on the sagittal plane. Evidence level: IV Case series.

## INTRODUCTION

The natural history of shoulder dislocation varies greatly according to the age when the injury occurs. In patients younger than 40 years of age, the recurrence of instability is relatively frequent and results from an injury in the labrum/anterior capsule complex. In patients older than 40 years of age, recurrence of instability is infrequent, but dislocation is associated with injuries to the rotator cuff.[1] McLaughlin[2] explained these differences through the hypothesis that two dislocation supports exist: the anterior support (labrum–anterior capsule) and the posterior support (rotator cuff – greater tuberosity). An injury to the anterior support occurs in young patients, recurrence being frequent, whereas in older patients the posterior support is injured, with the recurrence being infrequent.

Although the instability in young patients has been widely discussed in the literature, there are few publications focusing on the dislocation in patients older than 60 years of age, and we did not find any paper that specifically analyzes recurrence at that age.

This paper shows that in patients older than 60 years of age, recurrence is due to an injury to the anterior support, and an isolated lesion to the posterior support is insufficient to cause recurrence.

The goal of this paper is to clarify the role of associated injuries with respect to loss of function and recurrence of dislocations in such patients.

## MATERIALS AND METHODS

During the period ranging from January 2002 to 2007, we received in our Emergency Department a total of 64 patients older than 60 years of age, presenting with a first episode of glenohumeral dislocation.

We conducted a retrospective descriptive study where we reviewed these patients’ background, checking that there was imaging evidence showing dislocations, and that these were not associated with fractures (except for glenoid rim fractures or isolated greater tuberosity fractures). All patients were given an appointment for a physical examination by the first author, and they accepted to be enrolled in the study.

Patients had to comply with the following criteria to be enrolled in our study:

1. First episode of dislocation at an age older than 60 years
2. One-year minimum follow-up
3. Not having prior surgeries in the dislocated shoulder
4. Not having fractures associated with the dislocation, except for glenoid rim fractures and isolated greater tuberosity fractures
5. Attending visits
6. Accepting the performance of at least one magnetic resonance imaging (MRI) study of the shoulder in question

All patients, who were alive, (58), were given an appointment for visit. All patients included in the study signed a consent form, and accepted performance of imaging tests. No additional follow-up was performed for nonattendees.

The list of excluded patients and the causes for exclusion are shown on Table 1.

**Table 1:** Patients excluded from initial recruitment

Death	6
Fail to attend the visit	15
Fail to perform MRI	12
Previously operated shoulder	2

Total	35

MRI: Magnetic resonance imaging

Following these criteria, we enrolled 29 patients in the study, out of 64 patients. The 29 patients enrolled in this study who suffered a first glenohumeral dislocation at an age older than 60 years were referred to the Clinic and accepted to have an MRI study done on the affected shoulder.

1. We call the following structures the “anterior support:”
2. Anterior labrum
3. Subscapular tendon
4. Anterior rim of the glenoid cavity
5. We call the following structures the “posterior support”
6. Supraspinatus tendon
7. Infraspinatus tendon
8. Greater tuberosity

### Imaging assessment

**Figure 1:**
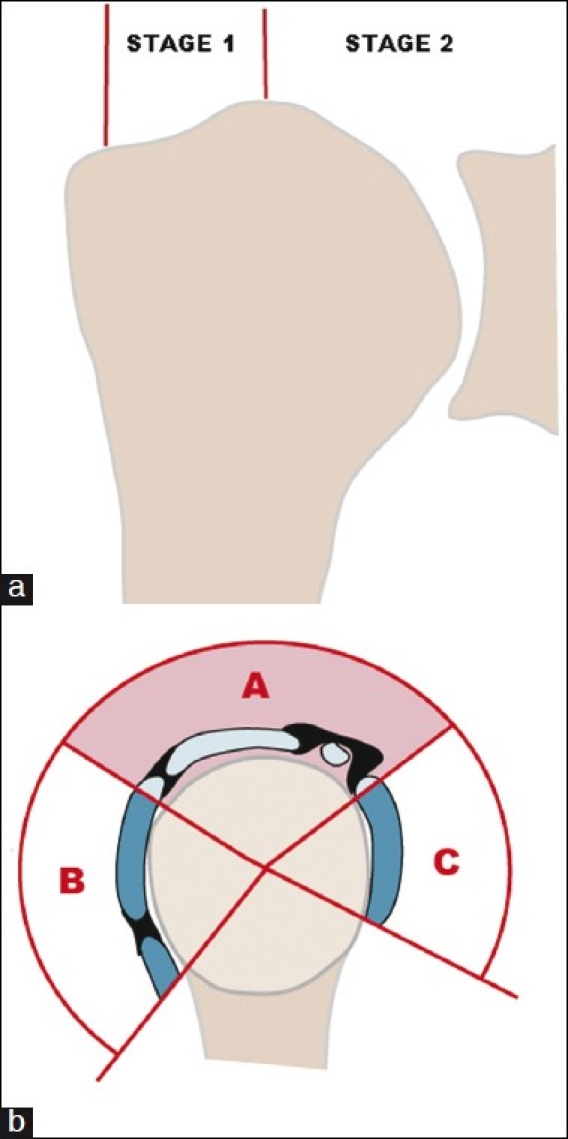
(a) Cuff involvement on the coronal plane. In Stage I, the cuff edge is distal to the highest point of the humeral head. In Stage II, the torn end of the cuff is medial to that point. (b) Cuff involvement on the sagittal plane, depending on the involvement of the supraspinatus/rotator interval (A), the infraspinatus/teres minor (B), or the subscapular tendon (C)

To assess the lesion degree of the rotator cuff by MRI images, we classified fractures according to their extension on the coronal plane (Stages 1 and 2) and on the sagittal plane (infraspinatus (B), supraspinatus (A), and/or subscapular (C) involvement), this being a modification to the classification utilized by other authors[3] [Figure 1]. Just full-thickness tears were considered in this study. Partial-thickness tears were not included.

For the assessment of fat tissue atrophy, we used Goutalier’s classification.[4] Imaging assessment was performed by a single musculoskeletal radiologist, coauthor of the paper, who knew neither the functional impact nor the number of dislocations suffered by the patient.

### Functional assessment

The shoulder’s functional assessment was conducted by means of the Constant test.[5] A Rapala® dynamometer, with a capacity of up to 25 kg and digital determination, was used to measure strength.

### Statistical analysis

In the univariable analysis, if variables are continuous they are described as mean and standard deviation (SD), and if they are qualitative, as ratios/percentages. For the bivariable analysis, the χ^2^ test or Fisher’s exact test was used (according to application conditions) for qualitative variables, or Student’s t-test for continuous variables that followed a normal distribution. The strength of the association between the dependent variable and the remaining independent variables was obtained by odds ratio estimated from the contingency tables and was expressed by the confidence interval (95%). The statistical analysis was performed with the SPSS, version 12.0 (SPSS Inc., 2003) statistical package, for Windows.

## RESULTS

All dislocations were anterior. All patients were right-handed. The mean follow-up from the first dislocation episode to the assessment in the clinic was of 27 months (range 13–59 months). The mean age of these patients at the moment of dislocation was 72.68 years (range 61–93 years).

A total of 9 (31.03%) patients had recurrent dislocation (more than one episode) [Table 2]. Four of these recurrent cases required reconstructive surgery to maintain joint stability. These four patients had suffered more than two dislocations when they underwent reconstructive surgery [Table 3].

**Table 2:** Relationship among recurrence, age, gender, and support involved

Recurrence	Number of cases	Age (years)	Gender (male/female)	Anterior support	Posterior support	Both support	Neither support
No	20	72	7/13	2	8	5	5
Yes	9	73	3/6	1	0	8	0

**Table 3:** List of patients with a recurrent shoulder dislocation

Case number	Gender	Age	Number of dislocations	Reconstructive surgery
2	F	74	7	Latarjet
4	F	82	2	No
6	F	67	9	HAGL reconstruction
10	F	93	4	HAGL reconstruction
11	F	67	2	No
16	M	61	2	No
18	M	64	10	Rotator cuff repair
19	M	70	2	No
21	F	84	2	No

F: Female; M: Male; HAGL: Humeral avulsion of the glenohumeral ligament

### Relationship between injury support and recurrence

Analysis of contingency tables that relate the affected support and recurrence shows a statistically significant relationship for the involvement of the anterior support (*P*=0.001; OR 34.2; IC_95%_ 1.74–673.73). In contrast, the posterior support involvement did not show statistical significance in relation to recurrence (*P*=0.37; OR 4.3; IC_95%_0.44–41.82). The involvement of both supports has demonstrated statistical significance with respect to recurrence (*P*=0.0033), even though recurrence has not occurred in any case in which the anterior support was not affected.

When the anterior support is affected, the injuries that tend to cause relapse are the anterior labrum injury and the injury of the anterior glenoid rim. A subscapular injury is not related to recurrence [Table 4].

**Table 4:** Injuries that affect the anterior support

Type of injury	Number of cases	Recurrence		
		Yes	No	Percentage
Subscapular tear	3	0	3	0
Anterior labral tear	14	9	5	64.2
Anterior glenoid fracture	4	2	2	50

### Relationship among functional outcome, rotator cuff involvement, and recurrence

The global incidence of rotator cuff tears has been 75.86% (22 cases). The prevalence of the full-thickness rotator cuff tear was higher in patients who had recurrent dislocation (88.8%) than in those who suffered just one episode of dislocation (70%) [Table 5].

**Table 5:** Relationship between the rotator cuff tear and dislocation recurrence

	Rotator cuff tear	
	Yes	No
Recurrent dislocation	8	1
Single-episode dislocation	14	6

Total	22	7

**Figure 2:**
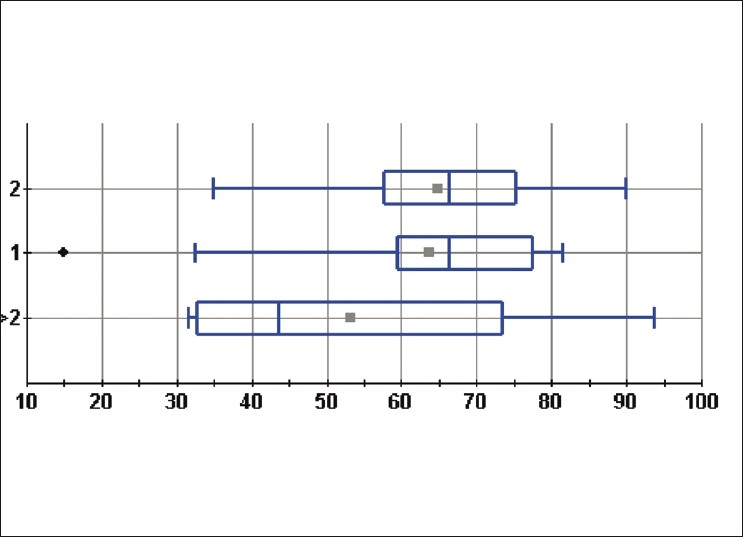
Constant score versus the number of dislocations. Vertical axis: number of dislocations: horizontal axis, Constant score

The functional outcome, according to Constant’s scale, was not significantly modified, as there was only one dislocation (Constant’s mean 63.6; DT 17.2) and it was recurrent (Constant’s mean 59.6; DT 23.6). Nonetheless, the function of the shoulder deteriorated when the patient suffered more than two dislocations [Figure 2].

The degree of the rotator cuff involvement on the coronal plane did not significantly influence the affected shoulder functional outcome. When the end of the supraspinatus tendon was in Stage I on the coronal plane [Figure 1], Constant’s mean was 57, DT 19.4; patients in Stage II showed a Constant mean of 58, DT 19.8.

The occurrence or nonoccurrence of a rotator cuff tear did have a significant impact on the shoulder function. Constant’s scale value was significantly higher when the rotator cuff was spared (mean 76.9; DT 8.6) than when it was torn (mean 57.7; DT 19.3).

Analyzing the sagittal plane, the extension of the tear anteriorly (subscapular) caused considerable deterioration of the functional outcome, whereas this detriment was not apparent when the tear extended posteriorly (infraspinatus) [Table 6].

**Table 6:** Relationship between rotator cuff involvement on the sagittal plane and Constant’s test

	Yes (M / SD)	No (M / SD)
Subscapular tear (anterior extension)	42/32.8	64.75/16.34
Infraspinatus tear (posterior extension)	59.4/24.5	63.34/17.63

M: Mean; SD: Standard deviation

## DISCUSSION

In this study, we have found that the main factor for the recurrence of dislocation in patients older than 60 years of age is anterior or combined (anterior or posterior) support’s involvement. The isolated injury of the posterior support is not related to recurrence. Our findings put into question the role of the posterior support in a dislocation’s recurrence in the adult population, the theory being advocated by McLaughlin.[2] The main lesion leading to recurrence in elderly patients is the glenoid rim and/or anterior rim injury. The frequent accompanying injury of the posterior support may deteriorate the functional outcome, but it is not the main cause of recurrence. The important role of the anterior support in recurrence has already been emphasized by other authors.[6] But which of the anterior support components cause recurrence? Araghi[6] reports 100% capsulolabral injuries in 11 patients older than 40 years of age with relapsing dislocations. However, Neviaser[7] does not find the Bankart lesion but anterior capsule and subscapular tears in 11 patients older than 50 years of age with recurrent dislocations. In our series, all patients who suffered a recurrent dislocation presented a Bankart lesion in MRI. This discrepancy is probable due to the method used for diagnosis (plain arthrography,[1] MRI or surgery.[6] It is also probable that performing an arthro-MRI may increase diagnosis of anterior capsule lesions that may be overlooked when a contrast agent is not used.

The extension of the rotator cuff tear should be evaluated carefully on the sagittal plane. The extension of the tear on the coronal plane did not correlate with the functional outcome, probably due to the fact that the coronal view could not see how many tendons were involved. The extension of the tear on the sagittal plane correlated with a significant decrease in the Constant score, especially in cases of anterior extension [Table 6]. This fact emphasizes the key role of the subscapularis tendon integrity. Nevertheless, we did not find any relationship between subscapularis involvement and recurrence [Table 4]. From our data, the subscapularis involvement had much more influence on the shoulder function than the recurrence rate.

Age (taking into account the population over the age of 60) is not related to ’recurrence’. However, recurrence in this age range is not a rare event in the literature (19–31%).[1,7–11] The pattern of recurrence probably differs from the one that young individuals present: in the Neviaser series,[7] recurrence is immediate, and the second episode of dislocation occurs by 1 week following the initial episode. This has not been analyzed in our series.

The function of the shoulder deteriorates when the patient suffers more than two dislocations. This is probably due to the extension of rotator cuff tear involvement.

Regarding cuff lesions in this patient population, we have drawn three conclusions:

1. The outcome is better when the cuff is spared following a dislocation
2. The degree of rotator cuff involvement on the coronal plane does not influence the functional outcome
3. The degree of rotator cuff involvement on the sagittal plane does influence the functional outcome, especially when the rupture extends anteriorly toward the subscapular tendon

It is not easy to know if the rotator cuff was torn during the dislocation episode, if it was torn before, or if there was an acute tear on a previous chronic tear. Nonetheless, if the imaging studies performed after the dislocation show that the cuff is not compromised, functional prognosis is promising. If MRI shows a cuff tear, we should carefully review the extension on the sagittal plane, often overlooked, because the anterior extension of the tear is a poor prognosis factor.

When these patients present a massive rupture of the cuff with an anterior support injury, which lesion should we repair? It is not the aim of this paper to know which type of repair regains the stability of the shoulder. Some authors indicate that repairing the Bankart lesion is not necessary in elderly patients, and that repairing the cuff is enough to stabilize the shoulder,[12] whereas others advocate in favor of repairing both supports.[13] We share the idea that both supports should be repaired as we know that we repair the posterior support to improve the function and that we repair the anterior support to improve the instability and function (in the case of subscapularis involvement).

This study has the following limitations: (1) a small number of patients due to difficulties in the recruitment and performance of complementary tests in this population group and (2) imaging study sensitivity. Performing an arthro-MRI with an intra-articular contrast agent would probably allow us to diagnose capsular injuries that may be overlooked in MRI without a contrast medium.

New multicenter studies are necessary to corroborate these findings.
